# Impact of Type 2 Diabetes Mellitus on Human Bone Marrow Stromal Cell Number and Phenotypic Characteristics

**DOI:** 10.3390/ijms21072476

**Published:** 2020-04-02

**Authors:** Féaron C. Cassidy, Ciara Shortiss, Colin G. Murphy, Stephen R. Kearns, William Curtin, Ciara De Buitléir, Timothy O’Brien, Cynthia M. Coleman

**Affiliations:** 1 College of Medicine, Nursing and Health Science, School of Medicine, Regenerative Medicine Institute (REMEDI), National University of Ireland Galway (NUI Galway), H91 FD82 Galway, Ireland; 2 Department of Trauma and Orthopaedics, Galway University Hospitals, H91 YR71 Galway, Ireland; 3 Saolta University Healthcare Group, Galway University Hospital, H91 YR71 Galway, Ireland; 4 CÚRAM Centre for Research in Medical Devices, College of Medicine, Nursing and Health Sciences, School of Medicine, NUI Galway, H91 FD82 Galway, Ireland

**Keywords:** type 2 diabetes mellitus, mesenchymal stem cells, mesenchymal stromal cells, adult stem cells, bone marrow stromal cells

## Abstract

Human bone marrow-derived mesenchymal stromal cells (MSCs) have been investigated in numerous disease settings involving impaired regeneration because of the crucial role they play in tissue maintenance and repair. Considering the number of comorbidities associated with type 2 diabetes mellitus (T2DM), the hypothesis that MSCs mediate these comorbidities via a reduction in their native maintenance and repair activities is an intriguing line of inquiry. Here, it is demonstrated that the number of bone marrow-derived MSCs in people with T2DM was reduced compared to that of age-matched control (AMC) donors and that this was due to a specific decrease in the number of MSCs with osteogenic capacity. There were no differences in MSC cell surface phenotype or in MSC expansion, differentiation, or angiogenic or migratory capacity from donors living with T2DM as compared to AMCs. These findings elucidate the basic biology of MSCs and their potential as mediators of diabetic comorbidities, especially osteopathies, and provide insight into donor choice for MSC-based clinical trials. This study suggests that any role of bone marrow MSCs as a mediator of T2DM comorbidity is likely due to a reduction in the osteoprogenitor population size and not due to a permanent alteration to the MSCs’ capacity to maintain tissue homeostasis through expansion and differentiation.

## 1. Introduction

Mesenchymal stromal cells (MSCs) are adult- or extraembryonic conceptus-derived multipotent progenitor cells. Theorised to play a crucial role in in vivo tissue repair and homeostasis, they have been strictly defined in vitro as having specific properties. These criteria, set by the International Society for Cellular Therapy (ISCT), include the ability to differentiate into osteoblasts, adipocytes and chondrocytes; plastic adherence in cell culture; and the presence and absence of a defined set of surface markers (CD73^+^, CD90^+^, CD105^+^, CD34^−^, CD45^−^, CD11b^−^ or CD14^−^, CD19^−^ or CD79α^−^, and HLA-DR^−^) [1]. As an in vivo population, MSCs are less well characterised but have been observed in histological sections using the ISCT-defined positive cell surface markers [2].

Because of the differentiation and immunoregulatory capacity of MSCs, their dysfunction has been proposed as the mediating mechanism in a wide variety of disease states. Shifts in the fate choice of MSCs, reduction in their overall proliferative capacity or in their differentiation to a particular cell type, as well as their role in mediating the activity of other cell populations have been demonstrated to play varying roles in disease progression [3,4,5].

In the context of type 2 diabetes mellitus (T2DM), whether T2DM may induce dysfunction in MSCs that reduces their capacity to mediate the effective homeostasis and the repair of bone is particularly pertinent. People living with T2DM are more likely to suffer from a fracture (relative risk 1.72) than the non-diabetic population, even when risk factors that predict likelihood of falling are taken into account [6]. Paradoxically, this increased risk in fracture is despite increased bone mineral density (BMD) in the T2DM population [7,8]. In the non-diabetic population, there is a strong negative correlation between BMD and fracture risk. Rodent models of T2DM have slender, weaker femurs, and extensive bone loss indicating qualitative changes (proposed to be mediated by advanced glycation end products (AGE)/receptor for advanced glycation end products (RAGE)) may play a role in BMD fracture risk discordance [9,10].

That MSCs are osteoblastic precursors in vivo [11] prioritises this cell type for investigation into whether T2DM can impact their ability to function appropriately in supporting bone homeostasis. Studies investigating this hypothesis have primarily taken place in exaggerated rodent models of T2DM and demonstrate reduced osteoblastogenesis (potentially via Notch2 signalling) and fewer osteoblast progenitors within the MSC population [10,12].

Very few studies have investigated the impact of T2DM on human bone marrow MSCs. Mahmoud et al. (2019) in their comprehensive review of the effects of diabetes mellitus (DM) on MSC function, report a total of 5 studies that assess the functional capacity of bone marrow-derived MSCs from people with T2DM [13], including two studies that focus on the differentiation of MSCs into insulin producing cells and three studies that assess outputs relating to MSC proliferative or differentiation capacity. Of those, Phadnis et al. and Liu et al. report reduced MSC proliferation in the T2DM context that is compounded by age and time since diagnosis [14,15]. Additionally, Ferland-Mccollough et al. (2018) describe increased adipogenic potential at the expense of osteogenesis in MSCs derived from people with T2DM [16]. Another study which exposed human MSCs to diabetic sera in culture demonstrated reduced viability [17].

Considering the hypothesis that dysfunctional MSCs are at the root of numerous degenerative diseases, it is somewhat unsurprising that they have been widely investigated as therapies. MSCs are currently being used in clinical trials to treat a wide range of pathologies, including comorbidities of T2DM. If MSCs from people with T2DM are dysfunctional, then their capacity to repair damaged organs either natively or as an autologous therapy may be depleted. Therefore, understanding the hypothesized T2DM-induced dysfunction in human MSCs is critical for two parallel branches of T2DM research: understanding the mechanistic aetiology of comorbidities (such as high fracture rates and deficient fracture repair) and progressing MSC-based regenerative medicine.

The aim of this study was to investigate whether there is a permanent impact on the MSC population and its functional capacity after exposure to the T2DM environment in vivo. Functional characteristics assessed were the number of MSCs present in bone marrow, proliferation, differentiation, angiogenic capacity, and migration. The findings demonstrate a reduced population of MSCs, specifically osteogenic progenitors, in bone marrow from people with T2DM but that these MSCs retain their in vitro functionality at levels comparable to that of age-matched controls (AMC). These results suggest that clinical studies intending to utilise MSCs to treat comorbidities of T2DM could consider autologous cell sources, bearing in mind that there is a T2DM-associated reduction in this cell type.

## 2. Results

The age range of the T2DM study cohort was 57–91. AMC control cohorts were selected to age-match the T2DM donor samples used for each assay; overall, the age range of AMC donors whose samples were used throughout the study was 51–89. The summary statistics of the donor age for the samples used in each assay are provided in Supplementary Table S1. The pharmacological profiles of a subset of 21 donors with T2DM were obtained, and 90% of these patients was recorded as being on at least one pharmacological agent for the treatment of T2DM. Of these, the most commonly prescribed was Metformin (84%), then Gliclazide (Diamicron, Diabrezide) (42%), and then gliptins (Sitagliptin, Linagliptin) (32%). 90% of the subset of T2DM donors pharmacologically profiled was on some form of blood pressure or heart medication, the most commonly prescribed being aspirin (47% of those on such medications were taking aspirin). Within this subset of 21 donors, 55% had recent HbA1c measurements of ≤48 mmol/mol and 85% of people had recent HbA1c of ≤58 mmol/mol, indicating an overall well-controlled population, taking into account the age of the donors. Those with HbA1c levels higher than 58 mmol/mol fell within a range of 70–80 mmol/mol.

Throughout, the bone marrow donors’ gender was assessed as a variable as there are conflicting reports in previous assessments regarding the impact of gender on MSC function [18,19]. Gender was not found to impact the number of or capacity of MSCs for any of the assays performed (Supplementary Figure S1), and therefore, the results comparing AMC to T2DM were not segregated by gender, allowing for increased statistical power in the reported analyses. Sample sizes for each assay, divided by gender, are provided in Supplementary Table S1.

### 2.1. Reduced Numbers of MSCs in the Bone Marrow of People Living With T2DM Compared to AMC, Due to a Specific Decrease in the Size of the Osteoblast-Committed Subpopulation

**Figure 1:**
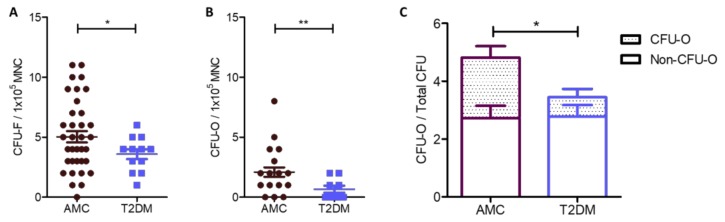
Quantification of the number of MSCs, detected as CFU-Fs, and those MSCs with native osteogenic potential (CFU-O) within whole marrow aspirates from AMC and T2DM cohorts. (**A**) Bone marrow from donors living with T2DM (*n* = 39) contained reduced numbers of CFU-Fs compared to bone marrow from the AMC cohort (*n* = 12). These data were normalised to MNC number and indicate the number of MSCs in the bone marrow is reduced in response to the T2DM environment. (**B**) CFU-O numbers in fresh bone marrow were identified by alkaline phosphatase staining and normalised to MNC number as per CFU-F. A reduction in the number of CFU-Os was recorded in the bone marrow in the T2DM cohort (*n* = 9) compared to that from the AMC cohort (*n* = 22). (**C**) In evaluating CFU-O number as a proportion of all CFUs in a donor bone marrow by two-way factorial analysis of deviance, diabetic status was observed to lead to a reduction in the proportion of CFU-Os. All data are displayed as mean ± SEM. * = *p* ≤ 0.05; ** = *p* ≤ 0.01.

A reduced number of MSCs was identified as a proportion of the mononuclear cell (MNC) population in colony-forming unit fibroblast (CFU-F) assays conducted using fresh whole bone marrow from people with T2DM (*n* = 12, 3.58 ± 0.42) compared to that from the AMC cohort (*n* = 39, 5.03 ± 0.47). These data are illustrated in Figure 1A and were analysed by Welch’s T-test due to unequal variance between groups (*p* = 0.0275).

MSC colonies were stained for alkaline phosphatase activity to indicate their osteogenic potential. Positively stained colonies were categorised as colony-forming unit osteoblasts (CFU-Os). CFU-O counts demonstrate the native osteoblastic capacity of MSCs and were generated without the addition of inductive differentiation factors. Bone marrow from AMC donors contained 2.09 ± 0.39 CFU-Os per 100,000 MNCs (*n* = 22), while marrow from donors living with T2DM (*n* = 9) contained 0.67 ± 0.29 CFU-Os per 100,000 MNCs (Figure 1B). A significantly lower number of CFU-O was therefore detected between the two groups when normalized to MNC number (Welch’s T test, *p* = 0.007, Figure 1B) or as a proportion of total CFU-F number (two-way analysis of deviance, *p* = 0.019, Figure 1C). Overall, 41 ± 5% of all CFU-Fs were CFU-Os in AMC marrow while 23% ± 6% of CFU-Fs in marrow from donors with T2DM were CFU-Os (Figure 1C).

CFU-Fs without osteogenic potential (i.e., the non-CFU-O CFU-Fs or nO-CFU-Fs) were compared and found to be almost identical in number between cohorts (AMC *n* = 22, 2.72 ± 0.42, compared to T2DM *n* = 9, 2.78 ± 0.40, T-test *p* = 0.944), demonstrating that the nO-CFU-Fs population remains unaffected by T2DM whilst the overall difference in CFU-F between the two cohorts results from the significant reduction in CFU-O numbers alone.

### 2.2. Cell Surface Characterisation and Cumulative Population Doublings of MSCs Unaffected by T2DM

**Figure 2:**
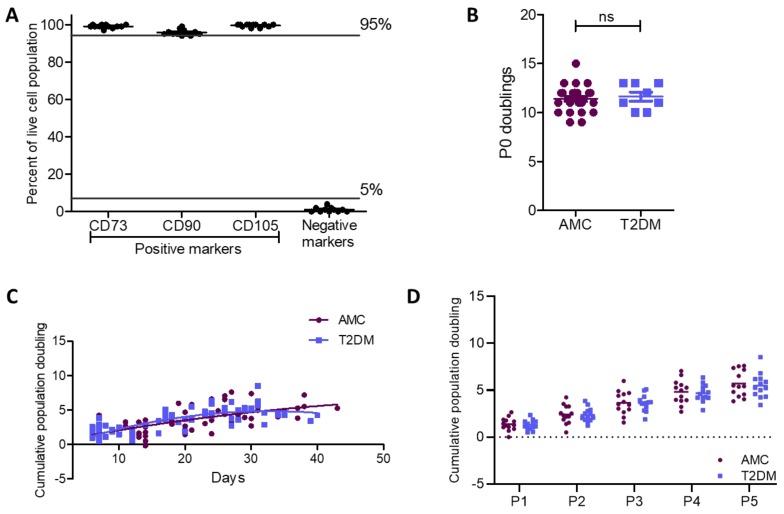
Cell surface phenotype and proliferative capacity were retained in MSCs from donors with T2DM as compared to AMCs. (**A**) Flow cytometry confirmed the population of bone marrow-derived primary cells isolated by plastic adherence as being MSCs due to their positive surface marker expression profile (CD73^+^, CD90^+^, and CD105^+^) and absence of CD34, CD45, CD11b or CD14, CD19 or CD79α, and HLA-DR (negative cocktail) expression (*n* = 12). (**B**) Population doubling at P0 was unaffected by the diabetic status of the donor (n = 28 for AMC and n = 8 for T2DM cohorts). (**C**) There was also no difference in doubling capacity between MSCs derived from individuals with T2DM or AMCs when cumulative population doublings per day were compared between the two groups (comparison of second-order polynomial nonlinear regression of the proliferation curve for each cohort of *n* = 13). (**D**) A 2-way ANOVA determined that there was no effect of T2DM on proliferative capacity per passage (P1–P5). All data are displayed as mean ± SEM. ns = not significant, i.e., *p* > 0.05.

Bone marrow-derived isolates were characterized by flow cytometry to confirm their cell-surface phenotype as MSCs as per Dominici et al. (2006) [1]. The cultures were 99.1% ± 0.3% positive for CD73, 95.8% ± 0.4% positive for CD90, 99.5% ± 0.2% positive for CD105, and 1% ± 0.4% positive for a set of negative markers, confirming their MSC identity (Figure 2A). Interestingly, late passage cultures demonstrated small but apparent differences in this characteristic expression pattern including a reduced CD90 population and increased negative marker population (Supplementary Figure S2A). Though changes to MSC surface marker profiles have been noted in late passage MSCs, the loss of CD90 which is considered a robust marker of MSC identity has not been previously recorded to our knowledge [20,21,22,23,24,25,26]. The presence of T2DM in the host did not affect this characteristic expression pattern in early passages (Figure 2A) or the altered expression at late passages (Supplementary Figure S2B,C).

MSCs were culture expanded in vitro from primary cells until the end of passage 5 to evaluate the proliferative capacity of these cultures. The number of MSCs was recorded at the initiation and completion of every passage (P0–P5). For P0 population doublings, the known number of CFU-F/100,000 MNC was used to calculate CFU-F/mL which provided a plating density for MSCs based on known plating volume (Figure 2B). During P0, MSC doubling in the AMC group (*n* = 28) was 11.4 ± 0.2 while that of donors with T2DM (*n* = 8) was 11.6 ± 0.5. A Mann–Whitney test illustrated the similarity between proliferative capacity of MSCs derived from donors from both the AMC and T2DM cohorts (*p* = 0.624).

Starting from P1 through to P5, cumulative doublings were recorded (*n* = 13 per cohort) and are presented in Figure 2 per number of days in culture (Figure 2C) and by passage number (Figure 2D). Cumulative doublings by MSCs in the AMC group were P1 = 1.36 ± 0.20, P2 = 2.42 ± 0.28, P3 = 3.66 ± 0.34, P4 = 4.82 ± 0.36, and P5 = 5.70 ± 0.39. Cumulative doublings for MSCs isolated from donors living with T2DM were comparable at P1 = 1.28 ± 0.15, P2 = 2.37 ± 0.20, P3 = 3.63 ± 0.24, P4 = 4.67 ± 0.25, and P5 = 5.49 ± 0.36. Statistical evaluation identified that there was no impact of T2DM on proliferative capacity per passage or over time, i.e., by day. The effect of DM status on doubling capacity per passage was assessed by two-way ANOVA with *p* = 0.779, while the doubling capacity per days in culture was calculated by nonlinear regression with comparison of the two curves (*p* = 0.448). MSC doubling capacity reduced with passage in accordance with previous studies [21]. This entry into a plateau phase is evident in Figure 2C.

### 2.3. Adipogenic and Osteogenic Differentiation Capacity not Permanently Unbalanced by T2DM

To assess the multi-lineage capacity of these primary cultures, MSCs from donors with or without T2DM were induced to differentiate into adipocytes and osteoblasts. There were no differences observed in differentiation capacity between MSCs from people with T2DM and AMC. 

**Figure 3:**
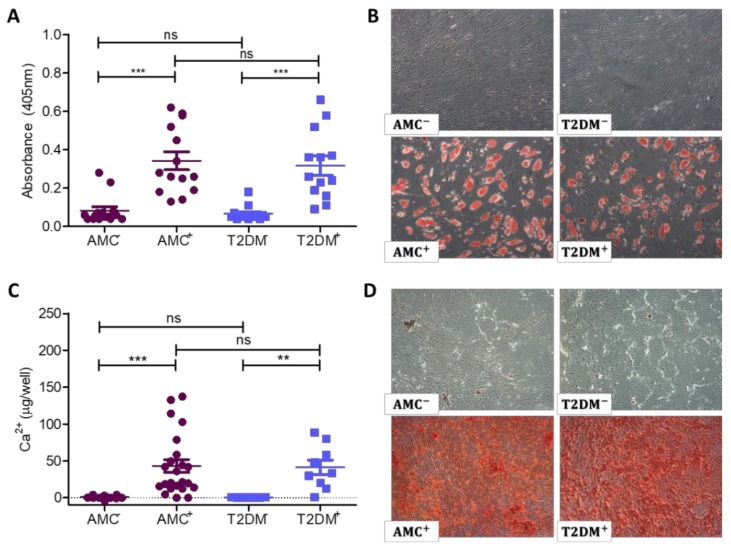
Adipogenic and osteogenic differentiation of MSC cultures. Undifferentiated cultures are identified as AMC^−^ or T2DM^−^, while differentiated cultures are identified as AMC^+^ or T2DM^+^. (**A**) MSC cultures were induced to form adipocytes, while undifferentiated cultures served as a control. Quantification of Oil Red O stain retention indicates a near absence of absorbed stain in undifferentiated cultures; then a statistically significantly enhanced retention in differentiated cultures. The presence of T2DM in the donor did not influence the culture’s capacity to undergo adipogenic differentiation (*n* = 13 AMC and *n* = 14 T2DM donor samples). (**B**) Representative images of each donor cohort demonstrating Oil Red O positive cells in differentiated cultures at the same frequency in cultures isolated from individuals with T2DM as in AMC cultures. Images obtained by 4× objective as described in methods. (**C**) Quantification of extracted calcium from osteogenically differentiated MSC cultures indicated their bone-forming potential. Calcium was present at negligible levels in undifferentiated AMC and T2DM control cultures but was significantly enhanced in differentiated cultures. There was no statistically significant impact of T2DM on the capacity of these cells to osteogenically differentiate (*n* = 23 for AMC and *n* = 9 for T2DM cohorts). (**D**) Staining of osteogenically differentiated cultures with Alizarin Red revealed the presence of calcium in the culture’s extracellular matrix. Undifferentiated controls were negative for Alizarin Red staining, while differentiated cultures retained stain. The distribution and intensity of the stain was comparable between donor cohorts. Images obtained by 4× objective as described in methods. All data are displayed as mean ± SEM. * = *p* ≤ 0.05; ** = *p* ≤ 0.01, ns = not significant, i.e., *p* > 0.05.

Adipogenically differentiated cultures were stained with Oil Red O to identify lipid vacuoles (Figure 3A,B). Undifferentiated cultures did not retain Oil Red O stain, while the lipid vacuoles in differentiated cultures stained red in the presence of Oil Red O. Photography by light microscope revealed no obvious differences in the quantity or size of stained cells between the two donor cohorts. The stain was then extracted, and its absorbance was read to quantify the amount of Oil Red O as a measure of the culture’s differentiation potential (Figure 3A). Extracts from undifferentiated AMC cultures (*n* = 13) had a mean absorbance of 0.082 ± 0.019, while those undifferentiated cultures from donors with T2DM (*n* = 14) had a mean absorbance of 0.066 ± 0.10, indicating negligible differentiation to adipocytes. In contrast, Oil Red O stained extracts from MSCs induced to adipogenically differentiate exhibited a mean absorbance of 0.342 + 0.046 in AMC cultures and 0.317 ± 0.049 in those derived from donors with T2DM. A 1-way ANOVA with a Tukey’s multiple comparison post hoc test demonstrated that both cohorts could be successfully induced to differentiate to adipocytes with a significant difference between the undifferentiated and differentiated MSC staining properties (*p* < 0.0001), while analysis of differentiated AMC absorbance measures compared to differentiated T2DM demonstrated no difference between groups (*p* = 0.7233).

**Figure 4:**
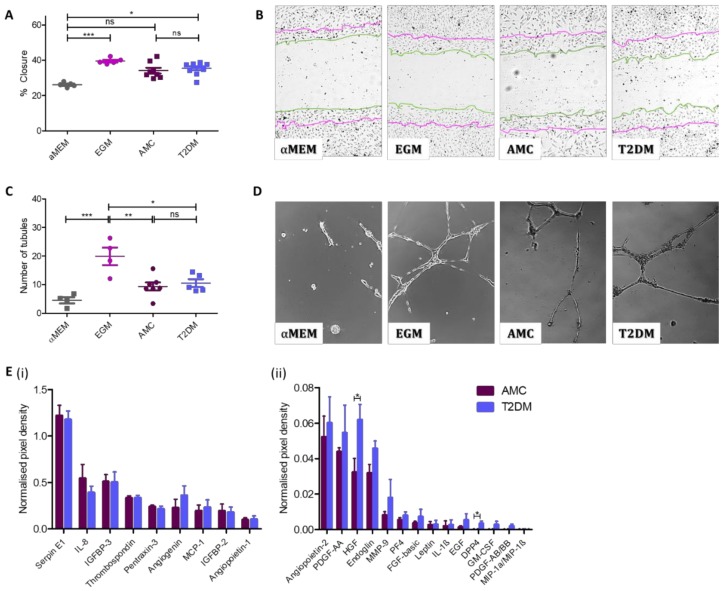
The presence of T2DM does not influence the capacity of MSC-secreted factors to support angiogenesis. The angiogenic capacity of MSCs to support migration of surrounding cells and to induce tubule formation was evaluated. (**A**) Quantification of HUVEC cell migration into a scratch in the HUVEC monolayer in the presence of MSC-conditioned medium indicated cells isolated from donors with (*n* = 10) or without T2DM (*n* = 8) were capable of stimulating HUVECs to close an in vitro wound. (**B**) Representative images display a notable decrease in the scratch width as a result of exposure to MSC-conditioned medium regardless of donor cohort. Images obtained by 10× objective as described in methods. Zero h scratch width is overlain onto the photograph taken at 8.5 h, with the 0 h outline traced in purple and the 8.5 h line outlined in green. (**C**) MSC-conditioned medium was capable of stimulating the formation of endothelial cell tubules regardless of its donor origin (AMC *n* = 7 and T2DM *n* = 5). (**D**) Representative images indicate the presence of long, branching tubules forming complete loops with a comparable morphology in cultures exposed to both types of MSC conditioned medium. Images obtained by 10× objective as described in methods. (**E**) Angiogenic protein levels in conditioned media at higher concentrations (**i**) and at lower concentrations (**ii**). Multiple T-test in R demonstrated increased levels of two secreted proteins (HGF and DPP4, mean ± SEM were 0.0322 ± 0.008 (AMC) and 0.0619 ± 0.009 (T2DM), and −0.002 ± 0.001 (AMC) and 0.003 ± 0.001 (T2DM), respectively), and no significant difference in levels where no statistics summary label is provided on the image (*n* = 4 per cohort). All data are displayed as mean ± SEM. * = *p* ≤ 0.05; ** = *p* ≤ 0.01, *** = *p* ≤ 0.001, ns = not significant, i.e., *p* > 0.05.

The calcium from osteogenically differentiated cultures was extracted and biochemically analysed (Figure 3C) or stained with Alizarin Red (Figure 3D) to identify the presence of calcium within the extracellular matrix. Undifferentiated control cultures exhibited negligible calcium deposition (AMC 0.922 ± 0.348 µg/well from *n* = 23 and T2DM 0.610 + 0.079 µg/well from *n* = 9) while MSCs induced to differentiate to the osteogenic lineage successfully formed calcium in significantly higher amounts (*p* < 0.0001) with AMC 43.19 ± 8.74 µg/well and T2DM 41.29 ± 9.99 µg/well, as displayed in Figure 4C. Analyses were performed by Kruskal–Wallis ANOVA with a post hoc Dunn’s multiple comparison test. There was no impact of T2DM on osteogenic differentiation capacity (*p* = 0.9021). Representative images (Figure 4D) show an even distribution of differentiated cells and similar staining intensity in both donor cohorts.

### 2.4. Angiogenic Capacity Comparable in MSCs Isolated From Donors With or Without T2DM

The scratch assay employed in this study mimics the endothelial cell migration during wound healing in vivo [27,28]. Here, a scratch assay was used to assess the capacity of MSC-secreted factors present in MSC-conditioned medium to stimulate endothelial cell (HUVEC) migration subsequent to creating a scratch through the HUVEC monolayer. In Figure 4A,B, the percentage of scratch closure is presented in cultures exposed to a negative control (αMEM), a positive control (endothelial growth medium (EGM)), or conditioned medium from AMC donor-derived MSCs or T2DM donor-derived MSCs. Non-stimulated scratch closure (αMEM treated) was 26.1% ± 0.58% (*n* = 5), while the EGM-treated scratches closed by 39.5% ± 0.76% (*n* = 5). Scratches treated with conditioned medium from AMC and T2DM donor samples both stimulated HUVEC migration over the negative control but displayed no differences in closure percentage between each other when analysed by 2-way Kruskal–Wallis ANOVA (nonparametric data). Those HUVECs treated with conditioned media from AMC donor MSCs closed by 34.1% ± 1.58% (*n* = 8) while those exposed to conditioned media from MSCs from donors with T2DM closed by 35.4% ± 1.08% (*n* = 10). Representative images of the HUVEC scratches at the end time-point show the level of scratch closure for each group (Figure 4B).

The tubule assay employed in this study mimics the endothelial cell differentiation during wound healing in vivo [28] by assessing the capacity of MSC-secreted factors to stimulate endothelial cell tubule formation over 18 h on a Matrigel substrate. Here, HUVEC cells were combined with either αMEM (negative control), EGM (positive control), or conditioned medium from either donor cohort before being plated on Matrigel. The mean number of resultant tubules identified in the negative control (*n* = 4) was 4.5 ± 1.1, significantly different to the 20 ± 3.1 tubules identified in the positive control (*n* = 4). The number of tubules created as a result of exposure to MSC-conditioned medium was comparable between the two MSC donor cohorts. In cultures exposed to AMC MSC-conditioned medium (*n* = 7), a mean of 9.4 ± 1.4 tubules formed, while in cultures exposed to MSC-conditioned medium from donors living with T2DM (*n* = 5), 10.5 ± 1.3 tubules formed. These data were normally distributed and assessed via ANOVA (Figure 4C,D).

The presence of angiogenic factors (angiogenin, angiopoietin-1, angiopoietin-2, DPP4, EGF, endoglin, fibroblast growth factor (FGF)-basic, GM-CSF, HGF, IGFBP-2, IGFBP-3, IL-1β, IL-8, leptin, MCP-1, MIP-1a/MIP-1β, MMP-9, PDGF-AA, PDGF-AB/BB, pentraxin-3, PF4, serpin E1, and thrombospondin) in MSC-conditioned medium was analysed by chemiarray (AMC *n* = 4, T2DM *n* = 4). The overall concentrations of angiogenesis-associated secreted proteins were comparable in the conditioned medium of both types of MSC cultures (Figure 4E). The exceptions were HGF and DPP4. Both HGF and DPP4 were observed at higher levels in the conditioned media of MSCs derived from people with T2DM than in that derived from MSCs from people in the AMC cohort.

### 2.5. Comparable Self-Mobility in T2DM and AMC Cohorts

**Figure 5:**
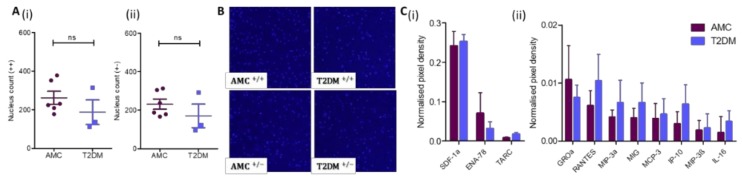
MSC mobility is not impacted by the diabetic status of the marrow donor. (**A**) As assayed through a transwell culture system, the (**i**) chemokinetic and (**ii**) chemotactic migration of MSCs from AMC (*n* = 6) and T2DM (*n* = 3) cohorts are comparable. (**B**) Representative images demonstrate the underside of a transwell with the migrated nuclei stained with Hoechst. (**C**) Chemokine levels in conditioned media at higher concentrations (**i**) and at lower concentrations (**ii**). Multiple T-test in R demonstrated no significant difference in levels (*n* = 4 per cohort). All data are displayed as mean + SEM, ns = not significant, i.e., *p* > 0.05.

MSC mobility is an indication of capacity to migrate to a site of injury. Here, MSC chemokinesis, random multidirectional movement, and chemotaxis, which is directed movement to an attractant, were assessed in a transwell system using serum as an attractant. Chemokinesis was assessed using a transwell system where serum was present in both the upper and lower chambers (Figure 5Ai,B). Wells containing MSCs derived from AMC donors (*n* = 6) had a mean of 261.8 + 33.6 cells on the underside of the transwell system as compared to those derived from donors with T2DM (*n* = 3) with a mean of 187.3 ± 63.7 cells migrating through the transwell. Analysis of the data by T-test revealed no significant difference between the two groups (*p* = 0.286). Chemotaxis was investigated, again using a transwell but with serum present only in the lower chamber (Figure 5Aii,B). In this scenario, a mean of 231.5 ± 26.3 AMC-derived cells (*n* = 6) were present on the underside of the transwell, while 170.3 ± 61.33 MSCs derived from donors living with T2DM (*n* = 3) migrated through the transwell. Again, analysis of these results by T-test (*p* = 0.308) did not reveal an affect due to the diabetic status of the donor.

Complimentary to these data, the presence and abundance of chemokines secreted by MSCs were profiled by chemiarray analysis (*n* = 4 donors per cohort). Chemokines (ENA-78, GROa, IL-16, IP-10, MCP-3, MIG, MIP-3a, MIP-3β, RANTES, SDF-1a, and TARC) were expressed at comparable levels in the conditioned medium of both types of MSC cultures and were not impacted by residing within the T2DM milieu (Figure 5C).

## 3. Discussion

Osteopathy is an increasingly recognized complication of T2DM. It was here hypothesized that T2DM impacts the bone marrow MSC (osteoblastic precursor) population, with potential implications for bone homeostasis in humans. This is in line with what has been observed in other complications associated with DM (reviewed in [29]) and in rodent models of T2DM [10,12]. Osteopathic comorbidities associated with T2DM include an increase in BMD [7], increased fracture risk [6,11] and delayed fracture repair [30].

It is important to note that the MSC isolation and cultivation methodologies, and tissue source can result in different functional capacities [31,32]. Additionally, animal and cell culture models must be assessed within their context, such that rodent models of DM often have additional metabolic abnormalities and can exaggerate the condition of a human population (especially those with well-controlled T2DM) [33], and that in vitro exposure of MSCs to high glucose is an incomplete model of DM.

In this study, MSCs from both T2DM and AMC human cohorts were successfully isolated from bone marrow and their number, proliferation capacity, differentiation potential, angiogenic capability, and migratory nature were assessed. The clinical importance of this lies not only in understanding the aetiology of T2DM bone dysfunction but also in that MSCs may be used to generate autologous therapies. The findings presented here demonstrate that, despite a decrease in osteogenic MSCs from people with T2DM at a population level, MSCs from people with T2DM have functionality across multiple reparatory-relevant assays at levels comparable to that of AMCs. These results suggest A) that T2DM-associated osteopathy is not related to the inherent nature of the progenitor cells but instead is likely a result of their numbers and B) that clinical studies intending to utilise MSCs to treat comorbidities of T2DM could consider autologous cell sources, bearing in mind reduced CFU-O proportion in calculating cell number for treatment.

Bone marrow-derived MSCs successfully isolated in this study as per the definition by Dominici et al. [1] were plastic adherent and fibroblastic in morphology. Similar to reports by others, these bone marrow-derived cells isolated from donors with T2DM displayed the characteristic ISCT-defined markers of MSCs [14,34,35,36]. MSCs at late passage lost the characteristic cell-surface expression pattern, a feature that was not related to T2DM status.

The quantity of MSCs present within a tissue will directly impact their capacity to maintain homeostasis, their ability to mount a repair response, and the capability to generate enough cells ex vivo for use as a cellular therapy. In this study, there were reduced quantities of CFU-Fs described in isolates from the T2DM cohort compared to those from donors from the AMC cohort, indicating a reduced number of MSCs residing within the bone marrow in response to T2DM.

The findings presented here demonstrate in human cells comparable results to rodent models in which the ex vivo culture of rodent bone marrow-derived MSCs in acute high glucose resulted in decreased colony forming capacity [37], and in which a rat model of DM contained fewer CFU-Fs in the bone marrow [10]. Together, these data suggest that rodent models of DM accurately reflect the human condition in terms of MSC population size.

However, these results are in contradiction to studies of adipose-derived progenitors from human donors with and without DM, where DM did not alter the yield of MSCs [9] or where increased yields were recorded [38]. However, the former study did not differentiate between T1DM and T2DM donors, and neither study demonstrated that the cells used in the study are MSCs according to the standard definition [1,38,39]. Therefore, the impact of T2DM status on adipose-derived MSCs remains unclear and it is unknown whether that population is affected by T2DM in accordance with the bone-marrow-derived population.

The ex vivo proliferative capacity of bone marrow-derived MSCs was found to be comparable in humans living with or without T2DM, indicating directly that T2DM does not impact MSC expansion potential. It should be noted that proliferative alterations could be masked by a corresponding shift in cell death or senescence, though the findings here are supported by the literature, primarily studies assessing human MSCs isolated from the adipose tissue [39,40]. In contrast, decreased proliferation and viability respectively were seen in expansion of rodent bone marrow-derived MSCs in acute high glucose culture [37] and in culturing bone marrow-derived MSCs from nondiabetic individuals with serum from donors with DM [17]. This indicates that any effects on the proliferative capacity of MSCs in response to DM may be in the MSC response to factors, including glucose, in their surrounding environment rather than permanent alterations to the cells’ capacity. These findings demonstrate that MSC isolates from bone marrow are capable of expansion to create an autologous therapy and supports a new hypothesis that T2DM does not decrease MSC self-renewal capacity.

The number of osteogenic progenitors within the marrow was investigated by CFU-O assays as an indication of the quantity of osteoblast precursors present in the bone marrow; 41% of the AMC MSCs had osteogenic potential while a significant decrease to 23% of MSCs with osteogenic potential was observed in cells isolated from donors with T2DM. This parallels previous reports of reduced CFU-Os in a rat model of diabetes [10] and indicates that having fewer MSCs with the capacity to support bone homeostasis may be causative of the osteopathies recorded in people with T2DM. Though overall MSC numbers were reduced, this was specifically due to a loss of CFU-O, with nO-CFU-F numbers remaining comparable between groups.

That human MSCs derived from donors with T2DM can be successfully differentiated in culture to osteoblasts via induction medium has been previously demonstrated for MSCs derived from both adipose [36,41] and bone marrow [16]. Overall, reports presented in the literature concerning the impact of DM on MSC osteogenic potential concur with the findings in this study that exposure to T2DM when within the bone marrow environment did not impede the osteogenic differentiation capacity of the MSCs when cultured with differentiation factors ex vivo [16,35]. However, artificial models of T2DM, such as exposure to high glucose, have shown both decreased osteogenic potential of adipose-derived MSCs [40] and increased osteogenic potential of bone marrow-derived MSCs [42].

In this investigation, MSCs from both AMC and T2DM successfully differentiated ex vivo to adipocytes, as demonstrated by histochemical staining and biochemical analysis. These data are in agreement with previous reports [39] and indicate that isolated cells retain their multipotent nature as per ISCT guidelines. Several publications have investigated the hypothesis that the in vivo multipotent nature of the MSC population shifts in a DM context to favour an adipogenic differentiation pathway in humans [16,43] and in a mouse model [44]. Indeed, work by Kim et al. and Ferland-McCollough et al. demonstrate an increase in marrow adiposity in association with T2DM and hypothesise this as the mechanism of impaired skeletal health in T2DM [16,33]. Notably, the findings here are in contrast to the in-depth study by Ferland-McCollough et al., which reported increased transcription of adipogenic regulatory factors and adipogenic potential in bone marrow-derived MSCs isolated from donors living with T2DM [16]. That study assessed a small sample size of adipogenically differentiated MSCs from the bone marrow of people with and without T2DM. The publication found a significant increase in the number of adipocytes present in cultures derived from people with T2DM. The discrepancy between those published findings and the data presented here may be due to differences in the methodology between the two studies. In Ferland-McCollough et al., adipocyte number was assessed by a cell counting method compared to biochemical analysis of lipid quantity here. Additionally, the methodologies utilised to expand the cultures were different (Ferland-McCollough et al. used 20% fetal bovine serum (FBS) without FGF for cell culture, while here, 10% FBS and 1 ng/mL FGF was used). Increased concentrations of serum improve adipogenic differentiation of progenitor cells [45], and the role of FGF is discussed below. Finally, the definitions of T2DM and AMC cohorts vary between the two studies. Here, we assessed the adipogenic capacity of MSCs from 13 people with T2DM with a mean age of 74. More than half of this cohort had HbA1c levels below 48 mmol/mol (i.e., very well-controlled T2DM). The cohort of four individuals used for differentiation assays in the study by Ferland-McCollough et al. had HbA1c of >48 mmol/mol, indicating cohort with less well-controlled T2DM. Additionally, the cohorts used in that study were of a younger age than in this study.

Collectively, these findings demonstrate that T2DM does not permanently alter the ability of MSCs to differentiate into adipocytes or osteoblasts. Although the in vivo exposure to the diabetic milieu may still impact MSC’s in situ propensity for differentiation, if hypothesised in vivo deficits are restored during the ex vivo expansion of MSCs (in supplemented media containing serum and FGF or by exposure to the osteogenic induction factors present in the osteogenesis assay), then this period of expansion in culture may prove beneficial to future cellular therapies.

Currently, the field of MSC function is transitioning away from the idea that resident MSCs directly differentiate into repair tissue following injury but that their paracrine signalling is of greater importance to repair injury and to modulate immune responses (reviewed by Watson et al.) [46]. Therefore, the paracrine angiogenic capacity of MSCs has been extensively explored with a view to creating a cellular therapy to treat the macro- and microvascular complications of DM as well as to understand the aetiology of the same [29]. Here, MSCs isolated from AMCs or from donors living with T2DM displayed a comparable capacity to induce an angiogenic response in endothelial cells. Similar results were published by two groups demonstrating that adipose-derived MSCs isolated from donors with or without DM can induce tubulogenesis equally [39,47]. It was similarly demonstrated that the diabetic status of the bone marrow donor did not impact the overall presence and concentration of angiogenic factors within the conditioned medium. HGF levels specifically were observed to be increased in the T2DM cohort, in line with previous reports of adipose-derived MSCs [47], and that increased HGF is a known predictor of T2DM [48]. However, the findings presented here conflict with those reported by Rezaie (2018), where the acute ex vivo exposure of nondiabetic bone marrow-derived MSCs to diabetic serum resulted in the downregulation of angiopoetin-1 and angiopoetin-2 protein levels [49]. Together, these findings indicate that the presence of T2DM in the donor does not impact the capacity for MSCs to support angiogenesis by secreting comparable levels of proangiogenic factors to those isolated from AMCs. Therefore, it is expected that aetiology of human T2DM-induced osteopathy is due to a pathology other than dysfunctional MSC-induced angiogenesis.

MSC chemokinesis and chemotaxis were also investigated using a transwell system as an indication of their capacity to migrate to a site of injury. The findings indicate that MSC migratory capacity was not impacted by their exposure to T2DM in situ. Previously published acute in vitro models of DM utilising high glucose or serum from people with T2DM have described a reduction in migratory capacity of bone marrow-derived MSCs [49] and adipose-derived MSCs [35]. Similarly, a study by Serena et al. indicated that T2DM inhibited the migratory capacity of adipose-derived MSCs from donors with T2DM unequally, depending upon the original tissue source (subcutaneous or visceral adipose tissue) [50]. Although the findings presented here contradict those in the literature, the experimental design is distinct, as here, bone marrow-derived MSCs have not been treated with glucose or other factors to replicate the DM environment. Therefore, we find that T2DM does not influence the capacity of bone marrow-derived MSCs in an ex vivo context, indicating their potential to respond to an injury stimulus and their migratory potential if administered therapeutically following culture expansion.

As well as their own migratory capacity, the chemokine content of MSC-conditioned medium was assessed to evaluate T2DM MSCs’ potential to recruit reparative cells to a sight of injury. It was found that MSCs from donors with T2DM and MSCs from AMCs secreted similar levels of SDF-1, GROa, RANTES, MIP-3a, MCP-3, IP-10, MIP-3β, and IL-16. It is well established in the literature that the secretome of MSCs is impacted by tissue source, inherent donor variability, and culture conditions [51], and this variability likely outweighs the impact of T2DM in this study. Together, these data indicate that MSCs from donors with T2DM are capable of movement to maintain bone homeostasis, migration to a site of injury, and ability to recruit reparative cells to the injury site. The similarities reported here demonstrate that MSCs from donors with T2DM likely have comparable in situ paracrine and chemotactic activities to that of MSCs derived from nondiabetic donors and should be considered as a potential tissue source when developing advanced cell-derived therapies such as extracellular vesical-based strategies.

The findings presented here are directly relevant to human T2DM-induced osteopathy as human bone marrow-derived MSCs chronically exposed to the T2DM milieu in situ were utilised. T2DM-associated osteopathy is therefore potentially linked to the reduced osteoprogenitor population in the bone marrow of people with T2DM and not as a result of an alteration to the proliferative or differentiation capacity of bone marrow-residing MSCs. These data indicate that there is no cause for concern of reduced function with autologous treatment in T2DM patients following ex vivo expansion, taking into account the reduced CFU-O population in this cohort. Studies focusing on determining markers to enrich the CFU-O subpopulation in isolated MSCs (such as in [52]) could aid in the use of autologous MSC treatments for those with T2DM in the future.

The criteria used to define the T2DM cohort are important and, here, were relatively unconstrained being that comorbidities were not recorded and that donors included those with very well-controlled, moderately controlled, and not well-controlled T2DM. With the intention of understanding the mechanism of T2DM-associated osteopathy and the potential for autologous use of MSCs as a cellular therapy, this study is reflective of the wider T2DM population. However, further investigations that compare the characteristics of those with higher HbA1c levels to people with well-controlled T2DM will be important moving forward. Being able to separate the effects of T2DM status from other metabolic disorders that it often associates with (including obesity) will be another important line of study in this area, and further measures including BMI would be useful for this purpose.

Though diligently designed, there are limitations inherent in this study as all of the described assays are conducted following ex vivo expansion and may not replicate the in vivo situation directly. Given that all of the characterization assays currently available require large numbers of cells, this limitation could not be avoided. Importantly, because the data presented in this work describe the in vitro culture experience of MSCs that have undergone expansion, the results reflect those used to create a cellular therapy, thereby informing translational scientists and clinicians alike. Further, as recent reports have demonstrated that exposure to FGF may restore the viability and proliferative capacity of MSCs exposed to the T2DM environment to that of a nondiabetic counterpart [36], its addition to expansion medium should be considered in studies moving forward. Therefore, the ability of MSCs derived from the bone marrow of donors with T2DM to function equally well across the tested assays is potentially due to a resetting of function during in vitro expansion or by an overriding of their native differentiation preference by the induction factors utilised in, for example, differentiation assays. Determining the impact of culture conditions is important for the development of MSCs as cellular therapeutics.

An alteration to the MSC population was here hypothesised as the mechanism for T2DM-associated osteopathy. This study found a reduced number of osteoprogenitors within the MSC population of bone marrow from donors with T2DM as compared to AMC. No other properties of the MSC population assayed were found to be altered in response to derivation from a T2DM environment. These included differentiation to the adipo- and osteo-lineages, proliferation, angiogenesis, and protein secretion profiles. Therefore, the results presented here propose a reduction in CFU-O number as a potentially important mechanism for T2DM-associated osteopathies and dually support the role of autologous MSC therapies in individuals with T2DM.

## 4. Materials and Methods

Throughout, the experiments were block organised to ensure donors from each cohort were distributed evenly across experiments for each assay. The experimental operator was blinded to the donor cohort throughout the assay and its analysis.

### 4.1. Bone Marrow Donors

AMC and T2DM donors were recruited for bone marrow donation under informed consent from those undergoing hip replacement surgery at one of two Galway University Hospitals (Merlin Park Hospital and University Hospital Galway). A patient information leaflet was provided to all participants. The protocol complied with the Declaration of Helsinki and was granted ethical approval by the Clinical Research Ethics Committee, Merlin Park Hospital (C.A. 1621, 3 November 2016).

Overall, the investigated populations (AMC and T2DM) are aged populations (summary statistics of the donors are provided in Supplementary Table S1) who likely display the typical comorbidities. The mean age range of the T2DM cohort was used to define the AMC population used for comparative analysis. Donor data are listed in Supplementary Table S1 for those donors whose samples were utilised in the current study. Donors with T1DM were excluded from this study.

Approximately 1–3 mL of bone marrow was obtained from the femoral neck upon femoral head removal by the surgical team. Only tissue that would otherwise have been discarded was retained for research purposes. The bone marrow was decanted into a tube containing 500 μL heparin (Wockhardt, Clonmel, Ireland), and MSCs were isolated within 3 days of surgery date.

Presence or absence of T2DM was recorded by the surgical team according to the patient’s medical record. Diagnosis was therefore always on an occasion previous to the surgery and made according to the American Diabetes Association (ADA) guidelines [53].

### 4.2. Mesenchymal Stromal Cell Isolation

Whole bone marrow was centrifuged, and the supernatant containing plasma and adipocytes was discarded. The loose pellets of MNCs, MSCs, and red blood cells were resuspended in a known volume of phosphate-buffered saline (PBS, Gibco via Biosciences, Dun Laoghaire, Ireland, 14190094), and the number of MNCs was counted. This solution (fresh bone marrow) was plated to isolate MSCs based on plastic adherence in tissue culture flasks and plates in expansion medium (α-MEM with GlutaMAX™, Gibco, 32561029) supplemented with 10% fetal bovine serum (FBS, HyClone via ThermoFisher, Ballycollen, Ireland SV30160.03), 1% antibiotic antimycotic solution (Merck, Dublin, Ireland, A5955), and 1 ng/mL recombinant human FGF-basic (Peprotech, London, UK, 100-18B)) at 37 °C with 5% CO_2_. The end of the first passage (P0) was determined upon the presence of mature, near-confluent colonies of MSCs. At this time point, MSCs were collected by lifting with 0.25% trypsin-Ethylenediaminetetraacetic acid (EDTA, Gibco, 25200056), resuspended in freezing medium (10% DMSO (Sigma, Arklow, Ireland, D2650), 90% FBS), and stored in a liquid nitrogen biobank.

### 4.3. Colony Forming Unit Assays

An aliquot of the fresh bone marrow (as above) was plated in triplicate in a 6-well plate and maintained for 8-10 days in expansion medium. The resultant CFU-Fs and CFU-Os were washed in PBS, preserved in 10% neutral buffered formalin (Sigma, HT501128) and stained with 0.8% crystal violet (Sigma, C0775) in methanol (Sigma, 179957) or stained for alkaline phosphatase activity with p-nitroblue tetrazolium chloride/5-bromo-4-chloro-3-indolyl phosphate (NBT/BCIP, Merck, B1911) without fixation respectively. Crystal violet-stained CFU-Fs were washed in tap water, and NBT/BCIP was pipetted from CFU-Os to remove excess stain. All stained colonies were counted using a brightfield microscope. Plating densities of <300,000 MNC per well of a 6-well plate were excluded from analyses subsequent to sensitivity testing of the ability of a subpopulation to accurately predict the overall population (*n* = 10/p(1 − p)).

### 4.4. Flow Cytometry

Flow cytometry characterisation was performed using the BD Biosciences Stemflow Human MSC Analysis Kit (BD, Limerick, Ireland, 562245) according to the manufacturer’s instructions. The cells isolated from marrow by plastic adherence were assessed by flow cytometry at the end of the first passage for the expression of MSC-defining markers. These markers were based on the ISCT criteria for identification of human BM-MSCs [1]. The negative cocktail was a PE-conjugated mixture of CD34, CD45, CD11b or CD14, CD19 or CD79α, and HLA-DR antibodies, and the positive cocktail consisted of individually conjugated CD73, CD90, and CD105 antibodies.

### 4.5. Cumulative Population Doublings

The number of MSCs present in the bone marrow was assessed by counting the number of CFU-F (clonogenic cells, i.e., MSCs) and then mathematically extrapolating from the volume of marrow used in the CFU assay to the total marrow volume. Selection of MSCs was based on plastic adherence. MSC count was expressed as CFU-F per 100,000 MNC at P0.

Subsequently, cumulative population doublings were calculated by plating a known number of cells at the start of a passage and by counting the total number of cells harvested at the end of each passage up to the end of P5. The number of days per passage was recorded, allowing for cumulative doublings to be recorded per passage and over time. Doublings were calculated as per the following:

(1) $$\mathrm{Doublings}=\log2\left(\frac{number\;of\;cells\;harvested}{number\;of\;cells\;plated}\right)$$

### 4.6. Osteogenic and Adipogenic Differentiation

Differentiation of confluent monolayer MSCs to adipocytes was induced using 1 μM dexamethasone (Merck, D4902), 10 μg/mL human recombinant insulin (Merck, 11376497001), 200 μM indomethacin (Merck, I7378), and 500 μM 3-Isobutyl-1-Methyl-Xanthine (MIX, Merck, I7018) in high glucose Dulbecco’s modified eagle medium (DMEM, Gibco, D5796), with 10% FBS and 1% penicillin-streptomycin (PS, Gibco, 15140-122). The cultures were incubated in this differentiation media for three three-day periods, alternated by one day incubations in maintenance media (αMEM, 10% FBS, 10 μg/mL insulin, 1% PS). At the completion of three cycles, the cultures were incubated in maintenance media for 7 days. Staining of lipid droplets was achieved through 0.18% Oil Red O (Sigma, O0625) after fixation in 10% neutral buffered formalin. The cells were then washed with 60% isopropanol (Sigma, 109827) and counter stained with Harris Modified Hematoxylin Solution (Merck, HHS16) for bright light microscope photographs. Photographs were taken using the 4x objective lens on an Olympus BX43 brightfield microscope fitted with HD Chrome camera (1/.8”) and 0.5x C-mount adapter. For quantitative analysis of Oil Red O, 99% isopropanol was used to extract the stain which was measured optically (absorbance at 490 nm) using a multimode plate reader (Victor X3, Perkin Elmer, Dublin, Ireland).

Differentiation of MSCs to osteoblasts was induced by treating an 80% confluent monolayer of MSCs with 1 nM dexamethasone, 100 μM ascorbic acid 2-phosphate (Merck, A8960), and 10 mM ß-glycerophosphate (Merck, G9422) in low-glucose DMEM (Merck, D6046) with 10% FBS and 1% PS. Control, undifferentiated cultures were maintained in expansion medium. The cultures were fed twice weekly for up to 17 days. The cells were then washed with PBS, and the resultant calcium was extracted by collecting the cell sheet in 0.5 M hydrochloric acid (Sigma, 1090581000) and by incubating overnight with agitation at 4 °C. Calcium quantification was carried out using the Stanbio Calcium CPC liquicolour kit (Stanbio via ThermoFisher, 0150250) as per the manufacturer’s instructions. Calcium staining was also performed with 2% Alizarin Red S (Merck, A5533) in water after fixation in ice cold 95% methanol. Photographs were obtained as per the adipogenically differentiated cells.

### 4.7. Scratch Assay

Conditioned media was generated from P2 donor MSCs when 80% confluent in a T175 flask. The cells were washed with PBS before being incubated overnight at 37 °C and 5% CO_2_ in αMEM containing 1% PS. Conditioned media was collected and stored at −80 °C. HUVECs at P8 were grown until 90% confluent in endothelial growth media (EGM, Lonza, Basel, Switzerland, CC3162); 80,000 cells were seeded per well of a 48-well plate and incubated for 24 h at 37 °C 5% CO_2_ until reaching 100% confluence. Eight replicate wells were created per MSC-conditioned media donor. A P200 tip was used to create a single defined scratch in each cell monolayer at the center of the well. The nonadherent cells were removed by washing gently with PBS before the addition of conditioned media to adherent cells. Scratches were photographed by automated bright field microscopy on a Cytation 1 Imaging Reader using the 10x objective lens (BioTek, with Gen5 Version 3.04 software, Swindon, UK) immediately after the addition of condition media (0 h) and after 8.5 h incubation at 37 °C 5% CO_2_. Image J (National Institutes of Health, Bethesda, USA) was used to measure the total area of each scratch. The percent closure of the scratch was calculated as follows:

(2) $$\%\;closure=100-(\frac{Area\;0\;h\;x\;100}{Area\;8.5\;h})$$

### 4.8. Matrigel Assays

Conditioned medium was generated as described in the scratch assay. P7 HUVECs were grown until 90% confluent in EGM. A 48-well plate was coated with 110 µL growth factor-reduced Matrigel (Corning via ThermoFisher, 734-1101) and left to solidify at 37 °C for 30 min. HUVECs were washed twice with PBS before being resuspended in MSC conditioned media and plated on the Matrigel coating at a concentration of 50,000 cells/well. Plates were incubated at 37 °C 5% CO_2_ for 18 h before being imaged using the 10× objective lens on Olympus BX43 brightfield microscope (specifications as per differentiation assays). Five images were acquired per well in a cross formation. Three wells were assayed per MSC donor. The number of tubules per image was counted by eye for each well.

### 4.9. Chemiarray Analysis of Secreted Proteins

Conditioned medium was generated as described in the scratch assay. The Proteome Profiler Human Cytokine Array Kit from R&D Systems (Abingdon, UK, ARY022B) was used to quantify relative levels of secreted proteins in conditioned media according to the manufacturer’s instructions.

### 4.10. Transwell Migration Assay

After two passages, MSC migration capacity was assayed. The cells were grown to 80% confluence in expansion media and then serum starved and FGF-deprived for 5 h before trypsinization. In a 24-well plate, MSCs were distributed such that 35,000 cells were contained in 200 μL of the appropriate media within 8-μm transwell inserts. 700 μL of media (with or without FBS) was loaded into the lower chamber. The cells were incubated for 24 h, after which the media was removed from the upper chamber. The transwells were fixed in 10% neutral buffered formalin for 10 min and washed in PBS, and loosely adherent cells were removed by wiping the upper chamber with a cotton swab. The adherent migrated cells were stained using 1:2000 diluted Hoesht 33342 (ThermoFisher, H1399) in 1% triton X (Sigma, X-100) for 30 min in the dark. The transwells were then washed three times in PBS and imaged on an Olympus Ix71 inverted microscope with Exfo X-Cite^TM^ 120 fluorescence light source. The resultant binary images were analyzed with Image J software with an adjusted threshold of 45–60 such that the number particles between 100–600 μm^2^ in size were quantified.

### 4.11. Statistical Analyses

All data were statistically assessed using Graphpad Prism (San Diego, USA) and R (version 3.5.1).

## Supplementary Materials

Supplementary materials can be found at https://www.mdpi.com/1422-0067/21/7/2476/s1.

## Abbreviations

AGE	Advanced glycation end products
AMC	Age-matched controls
BMI	Body mass index
BMD	Bone mineral density
CD	Cluster of differentiation
CFU-F	Colony-forming unit fibroblast
CFU-O	Colony-forming unit osteoblast
CO_2_	Carbon dioxide
DMEM	Dulbecco’s modified eagle medium
DM	Diabetes mellitus
DMSO	Dimethyl sulfoxide
EDTA	Ethylenediaminetetraacetic acid
EGM	Endothelial growth medium
FBS	Fetal bovine serum
FGF	Fibroblast growth factor
GDPR	General data protection regulation (EU)
HGF	Hepatocyte growth factor
HLA-DR	Human leukocyte antigen—DR isotype
HUVEC	Human umbilical vein endothelial cell
ISCT	International Society of Cell and Gene Therapy
MEM	Minimum essential medium
MIX	3-Isobutyl-1-Methyl-Xanthine
MNC	Mononuclear cell
MSC	Mesenchymal stromal cell
MTT	3-(4,5-dimethylthiazol-2-yl)-2,5-diphenyltetrazolium bromide
NBT/BCIP	p-nitroblue tetrazolium chloride/5-bromo-4-chloro-3-indolyl phosphate
P	Passage
PBS	Phosphate buffered saline
PE	Phycoerythrin
PS	Penicillin-Streptomycin
RAGE	Receptor for advanced glycation end products
SEM	Standard error of the mean
T2DM	Type 2 diabetes mellitus
