# Supplementary material for: Impact of Type 2 Diabetes Mellitus on Human Bone Marrow Stromal Cell Number and Phenotypic Characteristics
Source: Int J Mol Sci. 2020 Apr 2;21(7):2476. doi: 10.3390/ijms21072476 (PMC7177361; doi:10.3390/ijms21072476)
Supplement: Supplementary file 1 [file ijms-21-02476-s001.pdf]

Supplementary Materials: Supplementary materials can be found at www.mdpi.com/xxx/s1.

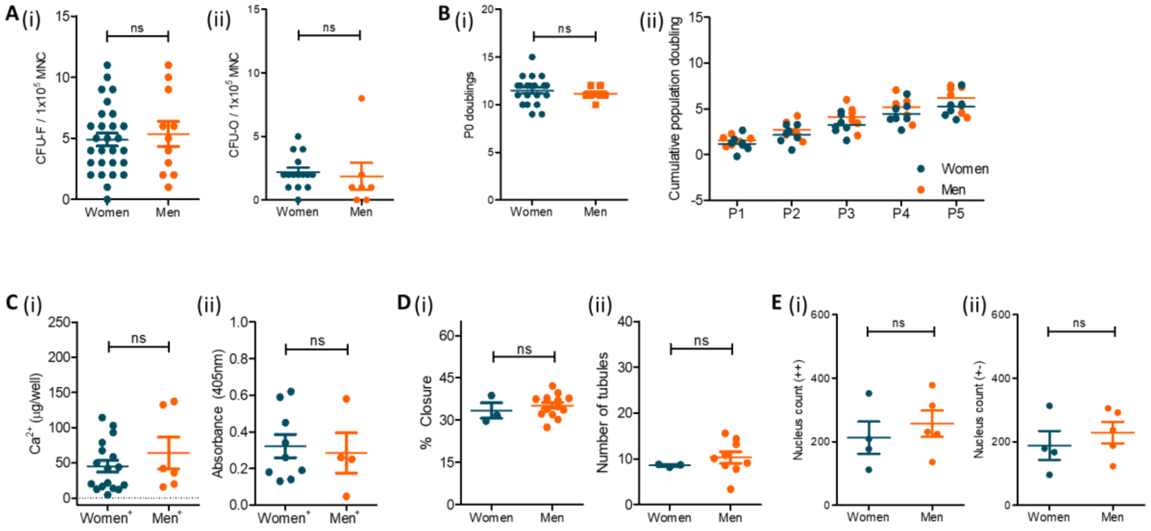

Supplementary Figure S1 Gender was not a significant variable for any of the assays performed in the study. (**A**) (i) CFU-F and (ii) CFU-O quantification, normalized to MNC number (CFU-F, T test, p = 0.6590 and CFU-O, Mann Whitney test, p = 0.1248). (**B**) (i) Proliferation and (ii) cumulative population doublings (P0, T test, p = 0.5626 and P1-5, 2-way ANOVA, p = 0.2725) (**C**) Differentiation [(i)Calcium deposition, Mann Whiteny test, p = 0.5518 and (ii) Oil Red O stain retention, T test, p = 0.7564 in differentiated cultures]. (**D**) Angiogenesis [(i) Scratch assay, T test, p = 0.4841, (ii) Matrigel assay, Welch's T test, p = 0.1984]. (**E**) Migration (i) chemokinesis, T test, p = 0.5174, (ii) chemotaxis T test, p = 0.4893).

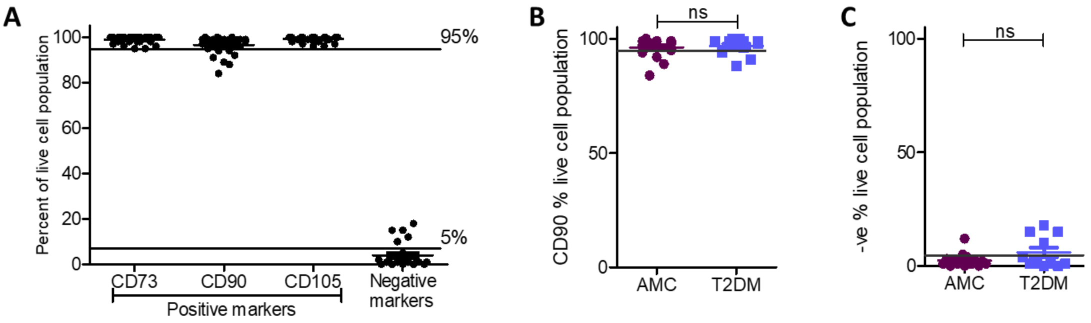

Supplementary Figure S2 Flow cytometry demonstrating (**A**,**B**) late passage (P4-P5) loss of positive MSC markers and (**A**,**C**) gain of negative MSC markers which were unaffected by T2DM status as assessed by Mann Whitney tests demonstrating (**B**) $p = 0.4305$, (**C**) $p = 0.5652$).

Supplementary Table S1: A summary of donor demographics, striated by assessment technique. For every assay, the number of biologic replicates is reported (n), followed by the number of women and men (w/m) within that group. The donors' collective mean age is reported ±SEM, as well as the minimum and maximum age of donors assessed in that assay.

Assay	Summary Statistics	AMC	T2DM
CFU-F	**n**	39	12
	(w/m)	27/12	6/6
	**Age**	72±1	73±2
	(min/max)	59/87	59/89
CFU-O	**n**	22	9
	(w/m)	15/7	6/3
	**Age**	72±1	73±3
	(min/max)	59/83	59/89
P0 doublings	**n**	32	8
	(w/m)	23/9	4/4
	**Age**	72±1	74±3
	(min/max)	59/87	59/89
P1-5 doublings	**n**	13	14
	(w/m)	7/6	7/7
	**Age**	73±3	73±2
	(min/max)	56/89	57/91
Osteogenesis	**n**	23	9
	(w/m)	17/6	3/6
	**Age**	73±2	73±3
	(min/max)	51/87	57/91
Adipogenesis	**n**	16	14
	(w/m)	11/5	6/8
	**Age**	73±2	74±2
	(min/max)	61/83	59/91
Scratch assay	**n**	7	9
	(w/m)	2/5	2/7
	**Age**	72±2	75±2
	(min/max)	67/78	69/91
Tubule assay	**n**	7	5
	(w/m)	2/5	1/4
	**Age**	69±3	73±1
	(min/max)	59/78	71/75
Transwell assay	**n**	6	3
	(w/m)	3/3	1/2
	**Age**	66±5	72±2
	(min/max)	56/84	69/76

Gender was demonstrated to have no impact on any of the assays performed in this study, in agreement with previous studies in human bone marrow MSCs [1], despite rat MSCs demonstrating increased therapeutic efficacy in in vivo studies [2,3]. As neither age nor gender impacted MSC number or function, there is no indication from these results that allogeneic MSC transplant should preference donors of a particular age or gender.. Indeed, these findings indicate that autologous treatment, which is preferential for a myriad of other reasons (from ethical to immunological), could be considered. Although donor age has previously been shown to impact the number of MSCs residing within the bone marrow, this effect was bimodal with young donors (5–20 years old), having a higher number of CFU-Fs than adult donors (20–45 years old), rather than a correlative effect with

overall aging [4]. Similarly, CFU-Fs were observed at an increased frequency in isolates from subcutaneous adipose tissue from young adult donors compared to donors over the age of 50, while no difference was recorded between donors aged 50–60 compared to 60–70 or >70 years of age [5] or between young donors (5 years to 30 years) [6]. In this study, there was no correlation found between donor age and CFU-F number (linear regression revealed no correlation with $p = 0.656$ for AMC at $n = 39$ and $p = 0.208$ at $n = 12$). However, it should be noted that the entire cohort of this study are an aged population (56–91 years of age) and that differences between young donors and older donors are not assessed here. This finding is in agreement with a review by Ganguly et al. [7], which discussed that aging of MSCs in vitro by passaging has a much greater impact than the age of the donor.

Reference:

1. Siegel, G.; Kluba, T.; Hermanutz-Klein, U.; Bieback, K.; Northoff, H.; Schäfer, R. Phenotype, donor age and gender affect function of human bone marrow-derived mesenchymal stromal cells. *BMC Med*. **2013**, *11*, 146, doi:10.1186/1741-7015-11-146.
2. Yuan, J.; Yu, J.-X. Gender difference in the neuroprotective effect of rat bone marrow mesenchymal cells against hypoxia-induced apoptosis of retinal ganglion cells. *Neural Regen. Res*. **2016**, *11*, 846–853, doi:10.4103/1673-5374.182764.
3. Sammour, I.; Somashekar, S.; Huang, J.; Batlahally, S.; Breton, M.; Valasaki, K.; Khan, A.; Wu, S.; Young, K.C. The Effect of Gender on Mesenchymal Stem Cell (MSC) Efficacy in Neonatal Hyperoxia-Induced Lung Injury, *PLoS ONE* **2016**, *11*, e0164269, doi:10.1371/journal.pone.0164269.
4. Stolzing, A.; Jones, E.; McGonagle, D.; Scutt, A. Age-related changes in human bone marrow-derived mesenchymal stem cells: Consequences for cell therapies. *Mech. Ageing Dev.* **2008**, *129*, 163–173, doi:10.1016/j.mad.2007.12.002.
5. Marędziak, M.; Marycz, K.; Tomaszewski, K.A.; Kornicka, K.; Henry, B.M. The Influence of Aging on the Regenerative Potential of Human Adipose Derived Mesenchymal Stem Cells. *Stem Cells Int.* **2016**, *2016*, 1–15, doi:10.1155/2016/2152435.
6. Muraglia, A.; Cancedda, R.; Quarto, R. Clonal mesenchymal progenitors from human bone marrow differentiate in vitro according to a hierarchical model. *J. Cell Sci.* **2000**, *113 Pt 7*, 1161–1166. http://www.ncbi.nlm.nih.gov/pubmed/10704367 (accessed March 3, 2020).
7. Ganguly, P.; El-Jawhari, J.J.; Giannoudis, P.V.; Burska, A.N.; Ponchel, F.; Jones, E.A. Age-related Changes in Bone Marrow Mesenchymal Stromal Cells: A Potential Impact on Osteoporosis and Osteoarthritis Development. *Cell Transplant.* **2017**, *26*, 1520–1529. doi:10.1177/0963689717721201.
